# Entrustable Professional Activities and the Future of Competency Evaluation in Neurology Residency Training

**DOI:** 10.1212/NE9.0000000000200290

**Published:** 2026-01-08

**Authors:** Jorge Patino, Elisabeth Breese Marsh, Robin Ulep, Kathryn Xixis, Peter B. Kang, Rachel Marie E. Salas

**Affiliations:** 1 James J. and Joan A. Gardner Family Center for Parkinson's Disease and Movement Disorders, University of Cincinnati, OH;; 2 Department of Neurology, Johns Hopkins University, Baltimore, MD;; 3 Department of Neurology, Icahn School of Medicine at Mount Sinai, New York, NY;; 4 Department of Neurology, University of Virginia, Charlottesville; and; 5 Department of Neurology, University of Minnesota Medical School, Minneapolis.

Competency-based medical education (CBME) has been promoted as the standard for evaluating neurology residency programs over the past decade. The rubric for evaluating residents has taken different forms, the most well-known being the Accreditation Council for Graduate Medical Education (ACGME) Milestones, which were last revised in 2020.^1^ The Milestones represent the knowledge, skills, attitudes, and other attributes necessary to achieve competency. Although effective in documenting a resident's proficiency regarding a specific competency, Milestones fall short in understanding a trainee's ability to integrate multiple competencies and practice unsupervised in the postgraduate clinical setting.^2^ Therefore, entrustable professional activities (EPAs), which aim to integrate competencies to deliver care, were introduced to bridge this gap and transition from focusing on the learning process to emphasizing learning outcomes (Figure).

**Figure:**
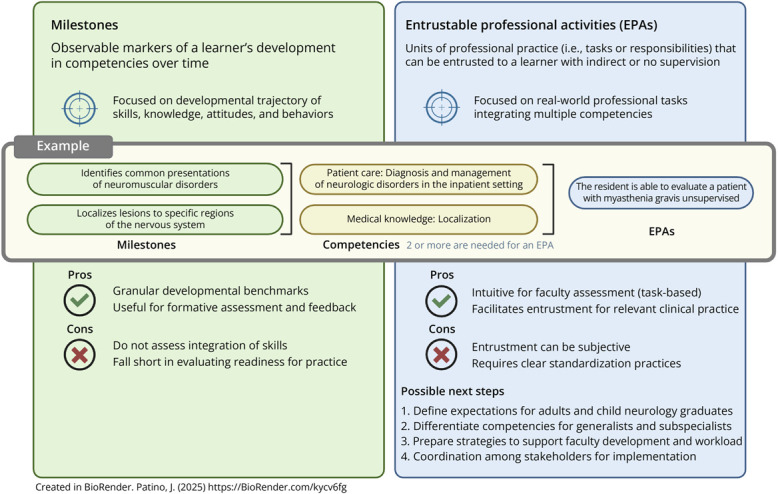
Comparison Between Milestones and Entrustable Professional Activities

The Association of American Medical Colleges released the Core EPAs for Entering Residency as a set of activities that residents are expected to perform on day one of residency. Recognizing its importance in neurology, the American Academy of Neurology (AAN) undergraduate education subcommittee published an article outlining the steps to develop EPAs for students participating in the neurology clerkship.^2^ At the graduate level, EPAs have been developed and applied by psychiatry,^3^ general surgery,^4^ and pediatrics residency programs in the United States and adult and child neurology in Canada. Their experiences demonstrate that national-scale organization and synchronized work among administrators, faculty, and learners are necessary for successful implementation. Novel technologies, such as a centralized smartphone app for timely assessments and real-time electronic medical record data input by the trainees, can be leveraged to mitigate the possible impact on the evaluator's time.

Considering the need for assessing residents' readiness to practice, the American Board of Psychiatry and Neurology (ABPN) hosted a Crucial Issues Forum in April 2025, which included representatives from adult and child neurology and psychiatry to discuss strategies for improving assessments of core training outcomes. We recognize the importance of these spaces in planning strategies to strengthen CBME in the United States, as well as in identifying opportunities and challenges to achieve this goal. We propose the creation of a task force in coordination with stakeholders, including the ABPN, AAN, American Neurological Association, Child Neurology Society, and ACGME, as well as academic institutions involved in residency training. Such a task force will need to define the EPAs for neurology residency, create evaluation rubrics and methodologies, incorporate technological tools for data management, and discuss the practical implications of EPAs for training certification, which encompass protected time for faculty, faculty development, and other resources and support for the residency program. In neurology, defining the line between what an adult and child generalist and a subspecialist should diagnose and treat in the context of a rapidly evolving field, faculty workload, and variability across programs can represent challenges.^5^ For this, tight coordination and collaboration between stakeholders are essential. This viewpoint is a call to action for neurology educators to organize and provide their perspective on applying novel methodologies for successful outcome assessment.
